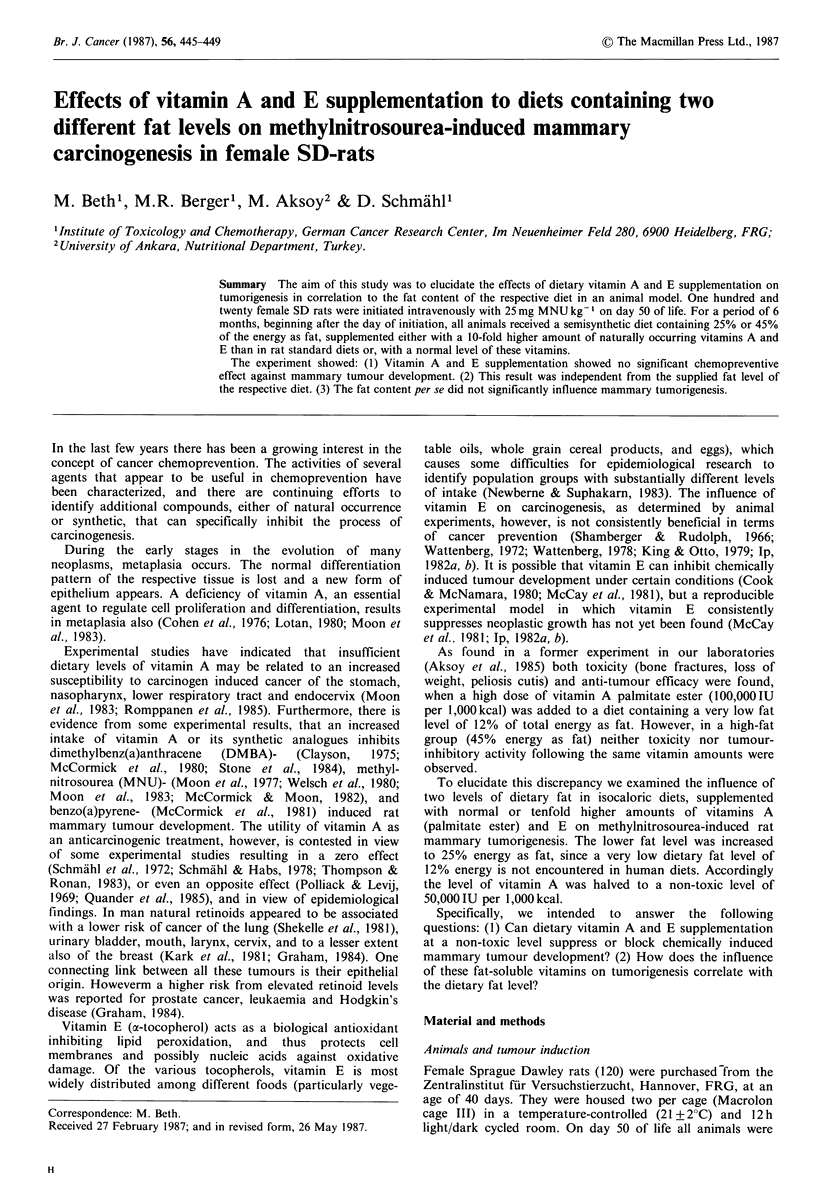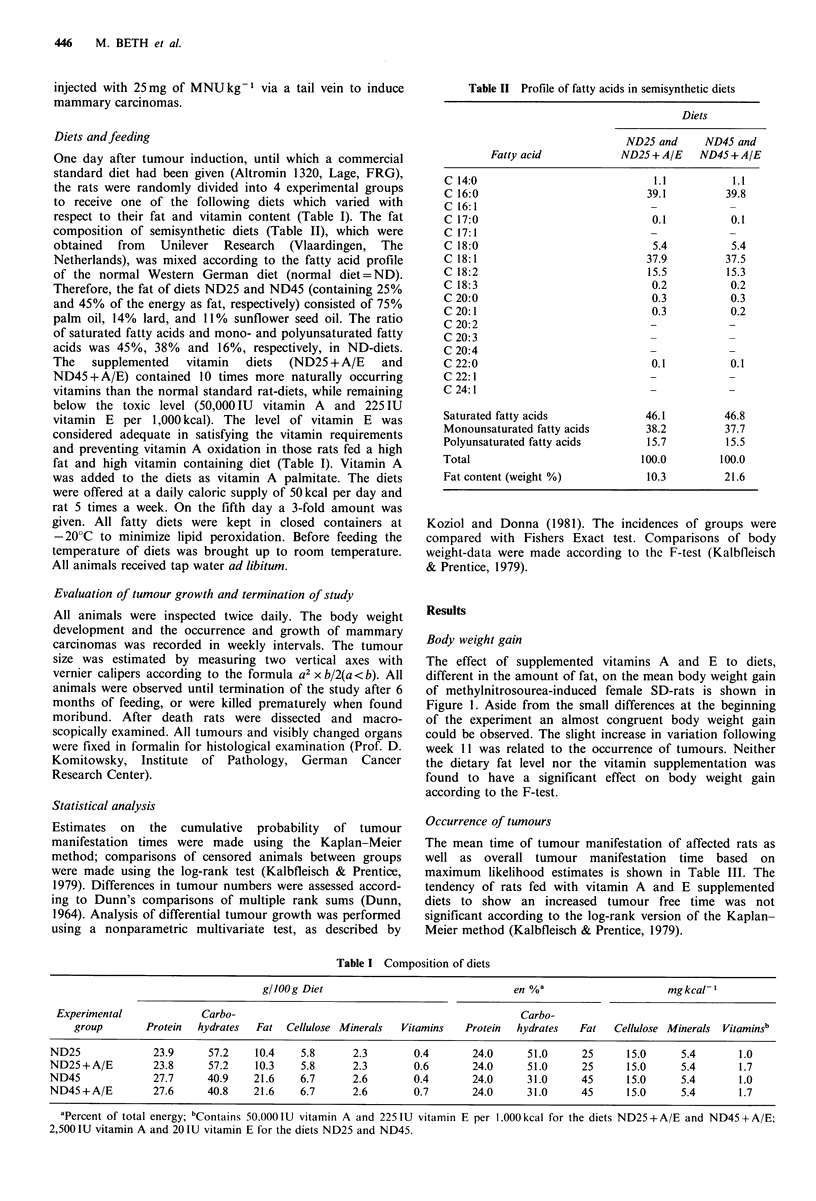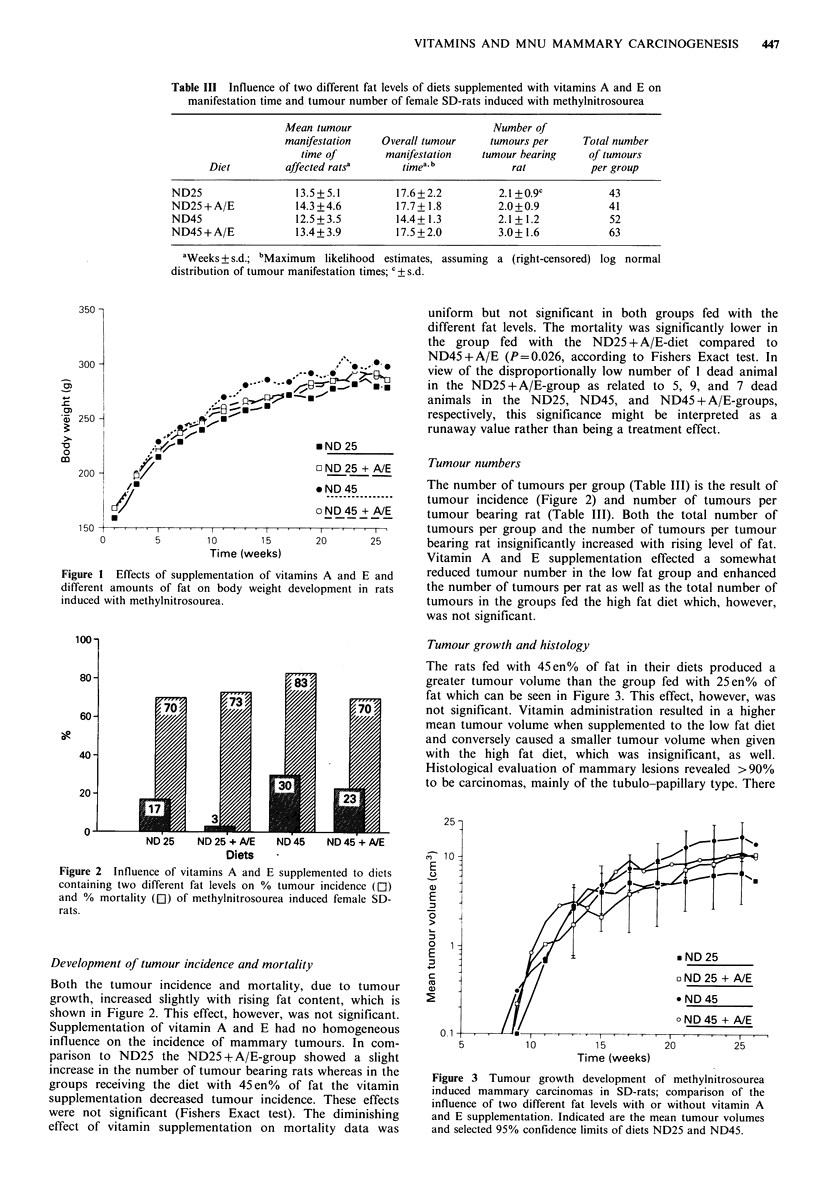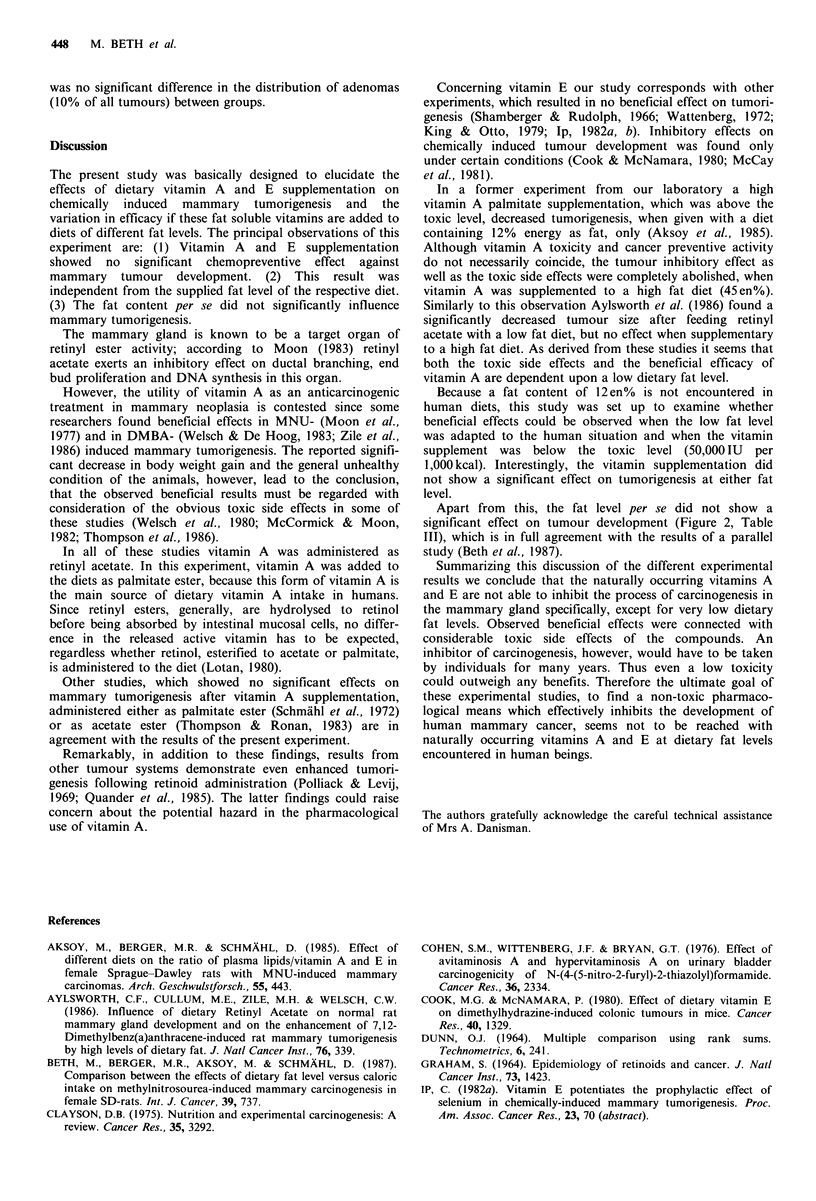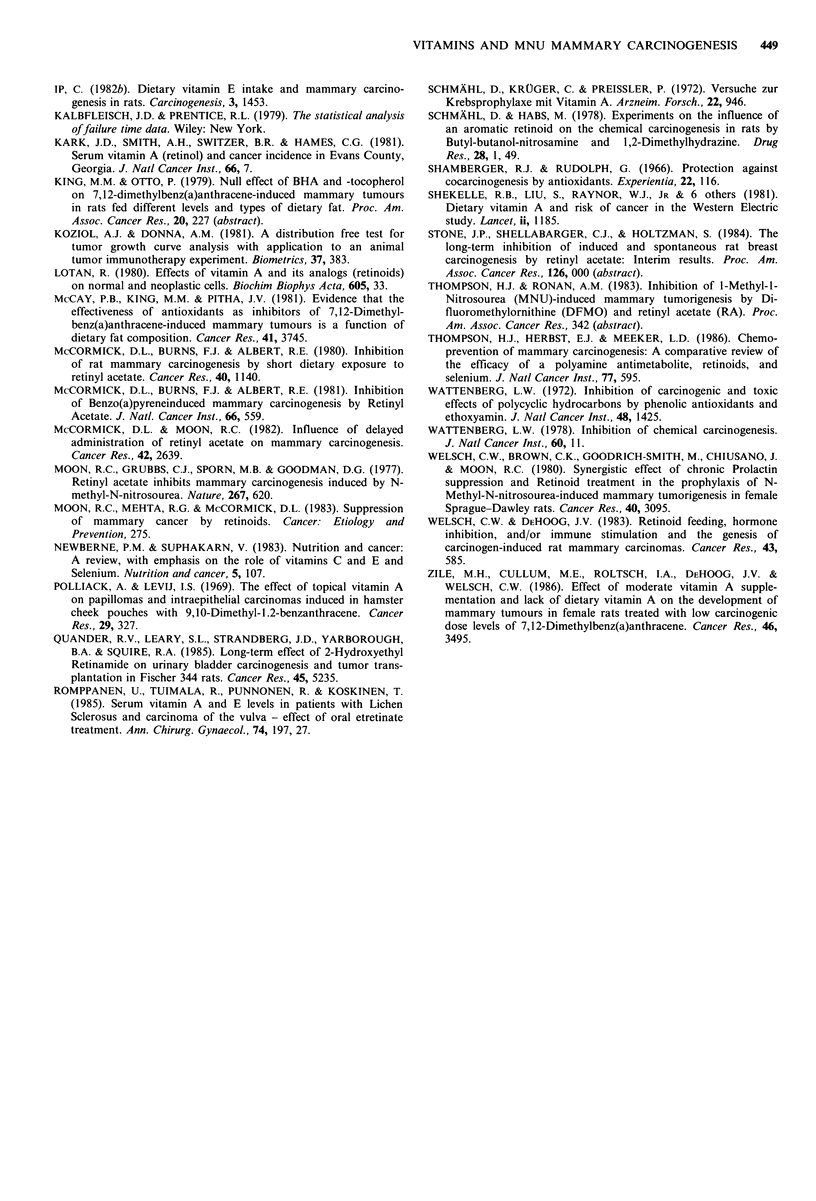# Effects of vitamin A and E supplementation to diets containing two different fat levels on methylnitrosourea-induced mammary carcinogenesis in female SD-rats.

**DOI:** 10.1038/bjc.1987.221

**Published:** 1987-10

**Authors:** M. Beth, M. R. Berger, M. Aksoy, D. Schmähl

**Affiliations:** Institute of Toxicology and Chemotherapy, German Cancer Research Center, Heidelberg.

## Abstract

The aim of this study was to elucidate the effects of dietary vitamin A and E supplementation on tumorigenesis in correlation to the fat content of the respective diet in an animal model. One hundred and twenty female SD rats were initiated intravenously with 25 mg MNU kg-1 on day 50 of life. For a period of 6 months, beginning after the day of initiation, all animals received a semisynthetic diet containing 25% or 45% of the energy as fat, supplemented either with a 10-fold higher amount of naturally occurring vitamins A and E than in rat standard diets or, with a normal level of these vitamins. The experiment showed: (1) Vitamin A and E supplementation showed no significant chemopreventive effect against mammary tumour development. (2) This result was independent from the supplied fat level of the respective diet. (3) The fat content per se did not significantly influence mammary tumorigenesis.


*Br. J. Cancer* (1987), **56**, 445–449
© The Macmillan Press Ltd., 1987

### Effects of vitamin A and E supplementation to diets containing two different fat levels on methylnitrosourea-induced mammary carcinogenesis in female SD-rats

M. Beth[1], M.R. Berger[1], M. Aksoy[2] & D. Schmähl[1]

[1]*Institute of Toxicology and Chemotherapy, German Cancer Research Center, Im Neuenheimer Feld 280, 6900 Heidelberg, FRG;* [2]*University of Ankara, Nutritional Department, Turkey.*

**Summary** The aim of this study was to elucidate the effects of dietary vitamin A and E supplementation on tumorigenesis in correlation to the fat content of the respective diet in an animal model. One hundred and twenty female SD rats were initiated intravenously with 25 mg MNU $kg^{-1}$ on day 50 of life. For a period of 6 months, beginning after the day of initiation, all animals received a semisynthetic diet containing 25% or 45% of the energy as fat, supplemented either with a 10-fold higher amount of naturally occurring vitamins A and E than in rat standard diets or, with a normal level of these vitamins.

The experiment showed: (1) Vitamin A and E supplementation showed no significant chemopreventive effect against mammary tumour development. (2) This result was independent from the supplied fat level of the respective diet. (3) The fat content *per se* did not significantly influence mammary tumorigenesis.

In the last few years there has been a growing interest in the concept of cancer chemoprevention. The activities of several agents that appear to be useful in chemoprevention have been characterized, and there are continuing efforts to identify additional compounds, either of natural occurrence or synthetic, that can specifically inhibit the process of carcinogenesis.

During the early stages in the evolution of many neoplasms, metaplasia occurs. The normal differentiation pattern of the respective tissue is lost and a new form of epithelium appears. A deficiency of vitamin A, an essential agent to regulate cell proliferation and differentiation, results in metaplasia also (Cohen *et al.*, 1976; Lotan, 1980; Moon *et al.*, 1983).

Experimental studies have indicated that insufficient dietary levels of vitamin A may be related to an increased susceptibility to carcinogen induced cancer of the stomach, nasopharynx, lower respiratory tract and endocervix (Moon *et al.*, 1983; Romppanen *et al.*, 1985). Furthermore, there is evidence from some experimental results, that an increased intake of vitamin A or its synthetic analogues inhibits dimethylbenz(a)anthracene (DMBA)- (Clayson, 1975; McCormick *et al.*, 1980; Stone *et al.*, 1984), methylnitrosourea (MNU)- (Moon *et al.*, 1977; Welsch *et al.*, 1980; Moon *et al.*, 1983; McCormick & Moon, 1982), and benzo(a)pyrene- (McCormick *et al.*, 1981) induced rat mammary tumour development. The utility of vitamin A as an anticarcinogenic treatment, however, is contested in view of some experimental studies resulting in a zero effect (Schmähl *et al.*, 1972; Schmähl & Habs, 1978; Thompson & Ronan, 1983), or even an opposite effect (Polliack & Levij, 1969; Quander *et al.*, 1985), and in view of epidemiological findings. In man natural retinoids appeared to be associated with a lower risk of cancer of the lung (Shekelle *et al.*, 1981), urinary bladder, mouth, larynx, cervix, and to a lesser extent also of the breast (Kark *et al.*, 1981; Graham, 1984). One connecting link between all these tumours is their epithelial origin. Howeverm a higher risk from elevated retinoid levels was reported for prostate cancer, leukaemia and Hodgkin's disease (Graham, 1984).

Vitamin E (α-tocopherol) acts as a biological antioxidant inhibiting lipid peroxidation, and thus protects cell membranes and possibly nucleic acids against oxidative damage. Of the various tocopherols, vitamin E is most widely distributed among different foods (particularly vegetable oils, whole grain cereal products, and eggs), which causes some difficulties for epidemiological research to identify population groups with substantially different levels of intake (Newberne & Suphakarn, 1983). The influence of vitamin E on carcinogenesis, as determined by animal experiments, however, is not consistently beneficial in terms of cancer prevention (Shamberger & Rudolph, 1966; Wattenberg, 1972; Wattenberg, 1978; King & Otto, 1979; Ip, 1982*a*, *b*). It is possible that vitamin E can inhibit chemically induced tumour development under certain conditions (Cook & McNamara, 1980; McCay *et al.*, 1981), but a reproducible experimental model in which vitamin E consistently suppresses neoplastic growth has not yet been found (McCay *et al.*, 1981; Ip, 1982*a*, *b*).

As found in a former experiment in our laboratories (Aksoy *et al.*, 1985) both toxicity (bone fractures, loss of weight, peliosis cutis) and anti-tumour efficacy were found, when a high dose of vitamin A palmitate ester (100,000 IU per 1,000 kcal) was added to a diet containing a very low fat level of 12% of total energy as fat. However, in a high-fat group (45% energy as fat) neither toxicity nor tumour-inhibitory activity following the same vitamin amounts were observed.

To elucidate this discrepancy we examined the influence of two levels of dietary fat in isocaloric diets, supplemented with normal or tenfold higher amounts of vitamins A (palmitate ester) and E on methylnitrosourea-induced rat mammary tumorigenesis. The lower fat level was increased to 25% energy as fat, since a very low dietary fat level of 12% energy is not encountered in human diets. Accordingly the level of vitamin A was halved to a non-toxic level of 50,000 IU per 1,000 kcal.

Specifically, we intended to answer the following questions: (1) Can dietary vitamin A and E supplementation at a non-toxic level suppress or block chemically induced mammary tumour development? (2) How does the influence of these fat-soluble vitamins on tumorigenesis correlate with the dietary fat level?

### Material and methods

### *Animals and tumour induction*

Female Sprague Dawley rats (120) were purchased from the Zentralinstitut für Versuchstierzucht, Hannover, FRG, at an age of 40 days. They were housed two per cage (Macrolon cage III) in a temperature-controlled (21±2°C) and 12 h light/dark cycled room. On day 50 of life all animals were

Correspondence: M. Beth.
Received 27 February 1987; and in revised form, 26 May 1987.

injected with 25 mg of MNU $kg^{-1}$ via a tail vein to induce mammary carcinomas.

*Diets and feeding*

One day after tumour induction, until which a commercial standard diet had been given (Altromin 1320, Lage, FRG), the rats were randomly divided into 4 experimental groups to receive one of the following diets which varied with respect to their fat and vitamin content (Table I). The fat composition of semisynthetic diets (Table II), which were obtained from Unilever Research (Vlaardingen, The Netherlands), was mixed according to the fatty acid profile of the normal Western German diet (normal diet = ND). Therefore, the fat of diets ND25 and ND45 (containing 25% and 45% of the energy as fat, respectively) consisted of 75% palm oil, 14% lard, and 11% sunflower seed oil. The ratio of saturated fatty acids and mono- and polyunsaturated fatty acids was 45%, 38% and 16%, respectively, in ND-diets. The supplemented vitamin diets (ND25 + A/E and ND45 + A/E) contained 10 times more naturally occurring vitamins than the normal standard rat-diets, while remaining below the toxic level (50,000 IU vitamin A and 225 IU vitamin E per 1,000 kcal). The level of vitamin E was considered adequate in satisfying the vitamin requirements and preventing vitamin A oxidation in those rats fed a high fat and high vitamin containing diet (Table I). Vitamin A was added to the diets as vitamin A palmitate. The diets were offered at a daily caloric supply of 50 kcal per day and rat 5 times a week. On the fifth day a 3-fold amount was given. All fatty diets were kept in closed containers at −20°C to minimize lipid peroxidation. Before feeding the temperature of diets was brought up to room temperature. All animals received tap water *ad libitum*.

*Evaluation of tumour growth and termination of study*

All animals were inspected twice daily. The body weight development and the occurrence and growth of mammary carcinomas was recorded in weekly intervals. The tumour size was estimated by measuring two vertical axes with vernier calipers according to the formula $a^2 \times b/2(a<b)$. All animals were observed until termination of the study after 6 months of feeding, or were killed prematurely when found moribund. After death rats were dissected and macroscopically examined. All tumours and visibly changed organs were fixed in formalin for histological examination (Prof. D. Komitowsky, Institute of Pathology, German Cancer Research Center).

*Statistical analysis*

Estimates on the cumulative probability of tumour manifestation times were made using the Kaplan–Meier method; comparisons of censored animals between groups were made using the log-rank test (Kalbfleisch & Prentice, 1979). Differences in tumour numbers were assessed according to Dunn's comparisons of multiple rank sums (Dunn, 1964). Analysis of differential tumour growth was performed using a nonparametric multivariate test, as described by Koziol and Donna (1981). The incidences of groups were compared with Fishers Exact test. Comparisons of body weight-data were made according to the F-test (Kalbfleisch & Prentice, 1979).

**Table II** Profile of fatty acids in semisynthetic diets

| Fatty acid | Diets: ND25 and ND25 + A/E | Diets: ND45 and ND45 + A/E |
|---|---|---|
| C 14:0 | 1.1 | 1.1 |
| C 16:0 | 39.1 | 39.8 |
| C 16:1 | – | – |
| C 17:0 | 0.1 | 0.1 |
| C 17:1 | – | – |
| C 18:0 | 5.4 | 5.4 |
| C 18:1 | 37.9 | 37.5 |
| C 18:2 | 15.5 | 15.3 |
| C 18:3 | 0.2 | 0.2 |
| C 20:0 | 0.3 | 0.3 |
| C 20:1 | 0.3 | 0.2 |
| C 20:2 | – | – |
| C 20:3 | – | – |
| C 20:4 | – | – |
| C 22:0 | 0.1 | 0.1 |
| C 22:1 | – | – |
| C 24:1 | – | – |
| Saturated fatty acids | 46.1 | 46.8 |
| Monounsaturated fatty acids | 38.2 | 37.7 |
| Polyunsaturated fatty acids | 15.7 | 15.5 |
| Total | 100.0 | 100.0 |
| Fat content (weight %) | 10.3 | 21.6 |

### Results

*Body weight gain*

The effect of supplemented vitamins A and E to diets, different in the amount of fat, on the mean body weight gain of methylnitrosourea-induced female SD-rats is shown in Figure 1. Aside from the small differences at the beginning of the experiment an almost congruent body weight gain could be observed. The slight increase in variation following week 11 was related to the occurrence of tumours. Neither the dietary fat level nor the vitamin supplementation was found to have a significant effect on body weight gain according to the F-test.

*Occurrence of tumours*

The mean time of tumour manifestation of affected rats as well as overall tumour manifestation time based on maximum likelihood estimates is shown in Table III. The tendency of rats fed with vitamin A and E supplemented diets to show an increased tumour free time was not significant according to the log-rank version of the Kaplan–Meier method (Kalbfleisch & Prentice, 1979).

**Table I** Composition of diets

| | g/100 g Diet | | | | | | en %[a] | | | mg kcal$^{-1}$ | | |
|---|---|---|---|---|---|---|---|---|---|---|---|---|
| Experimental group | Protein | Carbohydrates | Fat | Cellulose | Minerals | Vitamins | Protein | Carbohydrates | Fat | Cellulose | Minerals | Vitamins[b] |
| ND25 | 23.9 | 57.2 | 10.4 | 5.8 | 2.3 | 0.4 | 24.0 | 51.0 | 25 | 15.0 | 5.4 | 1.0 |
| ND25 + A/E | 23.8 | 57.2 | 10.3 | 5.8 | 2.3 | 0.6 | 24.0 | 51.0 | 25 | 15.0 | 5.4 | 1.7 |
| ND45 | 27.7 | 40.9 | 21.6 | 6.7 | 2.6 | 0.4 | 24.0 | 31.0 | 45 | 15.0 | 5.4 | 1.0 |
| ND45 + A/E | 27.6 | 40.8 | 21.6 | 6.7 | 2.6 | 0.7 | 24.0 | 31.0 | 45 | 15.0 | 5.4 | 1.7 |

[a]Percent of total energy; [b]Contains 50,000 IU vitamin A and 225 IU vitamin E per 1,000 kcal for the diets ND25 + A/E and ND45 + A/E; 2,500 IU vitamin A and 20 IU vitamin E for the diets ND25 and ND45.

**Table III** Influence of two different fat levels of diets supplemented with vitamins A and E on manifestation time and tumour number of female SD-rats induced with methylnitrosourea

| *Diet* | *Mean tumour manifestation time of affected rats*[a] | *Overall tumour manifestation time*[a,b] | *Number of tumours per tumour bearing rat* | *Total number of tumours per group* |
|---|---|---|---|---|
| ND25 | 13.5±5.1 | 17.6±2.2 | 2.1±0.9[c] | 43 |
| ND25+A/E | 14.3±4.6 | 17.7±1.8 | 2.0±0.9 | 41 |
| ND45 | 12.5±3.5 | 14.4±1.3 | 2.1±1.2 | 52 |
| ND45+A/E | 13.4±3.9 | 17.5±2.0 | 3.0±1.6 | 63 |

[a]Weeks±s.d.; [b]Maximum likelihood estimates, assuming a (right-censored) log normal distribution of tumour manifestation times; [c]±s.d.

**Figure 1** Effects of supplementation of vitamins A and E and different amounts of fat on body weight development in rats induced with methylnitrosourea.

**Figure 2** Influence of vitamins A and E supplemented to diets containing two different fat levels on % tumour incidence (□) and % mortality (▨) of methylnitrosourea induced female SD-rats.

### *Development of tumour incidence and mortality*

Both the tumour incidence and mortality, due to tumour growth, increased slightly with rising fat content, which is shown in Figure 2. This effect, however, was not significant. Supplementation of vitamin A and E had no homogeneous influence on the incidence of mammary tumours. In comparison to ND25 the ND25+A/E-group showed a slight increase in the number of tumour bearing rats whereas in the groups receiving the diet with 45en% of fat the vitamin supplementation decreased tumour incidence. These effects were not significant (Fishers Exact test). The diminishing effect of vitamin supplementation on mortality data was uniform but not significant in both groups fed with the different fat levels. The mortality was significantly lower in the group fed with the ND25+A/E-diet compared to ND45+A/E ($P=0.026$, according to Fishers Exact test. In view of the disproportionally low number of 1 dead animal in the ND25+A/E-group as related to 5, 9, and 7 dead animals in the ND25, ND45, and ND45+A/E-groups, respectively, this significance might be interpreted as a runaway value rather than being a treatment effect.

### *Tumour numbers*

The number of tumours per group (Table III) is the result of tumour incidence (Figure 2) and number of tumours per tumour bearing rat (Table III). Both the total number of tumours per group and the number of tumours per tumour bearing rat insignificantly increased with rising level of fat. Vitamin A and E supplementation effected a somewhat reduced tumour number in the low fat group and enhanced the number of tumours per rat as well as the total number of tumours in the groups fed the high fat diet which, however, was not significant.

### *Tumour growth and histology*

The rats fed with 45en% of fat in their diets produced a greater tumour volume than the group fed with 25en% of fat which can be seen in Figure 3. This effect, however, was not significant. Vitamin administration resulted in a higher mean tumour volume when supplemented to the low fat diet and conversely caused a smaller tumour volume when given with the high fat diet, which was insignificant, as well. Histological evaluation of mammary lesions revealed >90% to be carcinomas, mainly of the tubulo–papillary type. There

**Figure 3** Tumour growth development of methylnitrosourea induced mammary carcinomas in SD-rats; comparison of the influence of two different fat levels with or without vitamin A and E supplementation. Indicated are the mean tumour volumes and selected 95% confidence limits of diets ND25 and ND45.

was no significant difference in the distribution of adenomas (10% of all tumours) between groups.

### Discussion

The present study was basically designed to elucidate the effects of dietary vitamin A and E supplementation on chemically induced mammary tumorigenesis and the variation in efficacy if these fat soluble vitamins are added to diets of different fat levels. The principal observations of this experiment are: (1) Vitamin A and E supplementation showed no significant chemopreventive effect against mammary tumour development. (2) This result was independent from the supplied fat level of the respective diet. (3) The fat content *per se* did not significantly influence mammary tumorigenesis.

The mammary gland is known to be a target organ of retinyl ester activity; according to Moon (1983) retinyl acetate exerts an inhibitory effect on ductal branching, end bud proliferation and DNA synthesis in this organ.

However, the utility of vitamin A as an anticarcinogenic treatment in mammary neoplasia is contested since some researchers found beneficial effects in MNU- (Moon *et al.*, 1977) and in DMBA- (Welsch & De Hoog, 1983; Zile *et al.*, 1986) induced mammary tumorigenesis. The reported significant decrease in body weight gain and the general unhealthy condition of the animals, however, lead to the conclusion, that the observed beneficial results must be regarded with consideration of the obvious toxic side effects in some of these studies (Welsch *et al.*, 1980; McCormick & Moon, 1982; Thompson *et al.*, 1986).

In all of these studies vitamin A was administered as retinyl acetate. In this experiment, vitamin A was added to the diets as palmitate ester, because this form of vitamin A is the main source of dietary vitamin A intake in humans. Since retinyl esters, generally, are hydrolysed to retinol before being absorbed by intestinal mucosal cells, no difference in the released active vitamin has to be expected, regardless whether retinol, esterified to acetate or palmitate, is administered to the diet (Lotan, 1980).

Other studies, which showed no significant effects on mammary tumorigenesis after vitamin A supplementation, administered either as palmitate ester (Schmähl *et al.*, 1972) or as acetate ester (Thompson & Ronan, 1983) are in agreement with the results of the present experiment.

Remarkably, in addition to these findings, results from other tumour systems demonstrate even enhanced tumorigenesis following retinoid administration (Polliack & Levij, 1969; Quander *et al.*, 1985). The latter findings could raise concern about the potential hazard in the pharmacological use of vitamin A.

Concerning vitamin E our study corresponds with other experiments, which resulted in no beneficial effect on tumorigenesis (Shamberger & Rudolph, 1966; Wattenberg, 1972; King & Otto, 1979; Ip, 1982*a*, *b*). Inhibitory effects on chemically induced tumour development was found only under certain conditions (Cook & McNamara, 1980; McCay *et al.*, 1981).

In a former experiment from our laboratory a high vitamin A palmitate supplementation, which was above the toxic level, decreased tumorigenesis, when given with a diet containing 12% energy as fat, only (Aksoy *et al.*, 1985). Although vitamin A toxicity and cancer preventive activity do not necessarily coincide, the tumour inhibitory effect as well as the toxic side effects were completely abolished, when vitamin A was supplemented to a high fat diet (45 en%). Similarly to this observation Aylsworth *et al.* (1986) found a significantly decreased tumour size after feeding retinyl acetate with a low fat diet, but no effect when supplementary to a high fat diet. As derived from these studies it seems that both the toxic side effects and the beneficial efficacy of vitamin A are dependent upon a low dietary fat level.

Because a fat content of 12 en% is not encountered in human diets, this study was set up to examine whether beneficial effects could be observed when the low fat level was adapted to the human situation and when the vitamin supplement was below the toxic level (50,000 IU per 1,000 kcal). Interestingly, the vitamin supplementation did not show a significant effect on tumorigenesis at either fat level.

Apart from this, the fat level *per se* did not show a significant effect on tumour development (Figure 2, Table III), which is in full agreement with the results of a parallel study (Beth *et al.*, 1987).

Summarizing this discussion of the different experimental results we conclude that the naturally occurring vitamins A and E are not able to inhibit the process of carcinogenesis in the mammary gland specifically, except for very low dietary fat levels. Observed beneficial effects were connected with considerable toxic side effects of the compounds. An inhibitor of carcinogenesis, however, would have to be taken by individuals for many years. Thus even a low toxicity could outweigh any benefits. Therefore the ultimate goal of these experimental studies, to find a non-toxic pharmacological means which effectively inhibits the development of human mammary cancer, seems not to be reached with naturally occurring vitamins A and E at dietary fat levels encountered in human beings.

The authors gratefully acknowledge the careful technical assistance of Mrs A. Danisman.

### References

AKSOY, M., BERGER, M.R. & SCHMÄHL, D. (1985). Effect of different diets on the ratio of plasma lipids/vitamin A and E in female Sprague–Dawley rats with MNU-induced mammary carcinomas. *Arch. Geschwulstforsch.*, **55**, 443.

AYLSWORTH, C.F., CULLUM, M.E., ZILE, M.H. & WELSCH, C.W. (1986). Influence of dietary Retinyl Acetate on normal rat mammary gland development and on the enhancement of 7,12-Dimethylbenz(a)anthracene-induced rat mammary tumorigenesis by high levels of dietary fat. *J. Natl Cancer Inst.*, **76**, 339.

BETH, M., BERGER, M.R., AKSOY, M. & SCHMÄHL, D. (1987). Comparison between the effects of dietary fat level versus caloric intake on methylnitrosourea-induced mammary carcinogenesis in female SD-rats. *Int. J. Cancer*, **39**, 737.

CLAYSON, D.B. (1975). Nutrition and experimental carcinogenesis: A review. *Cancer Res.*, **35**, 3292.

COHEN, S.M., WITTENBERG, J.F. & BRYAN, G.T. (1976). Effect of avitaminosis A and hypervitaminosis A on urinary bladder carcinogenicity of N-(4-(5-nitro-2-furyl)-2-thiazolyl)formamide. *Cancer Res.*, **36**, 2334.

COOK, M.G. & McNAMARA, P. (1980). Effect of dietary vitamin E on dimethylhydrazine-induced colonic tumours in mice. *Cancer Res.*, **40**, 1329.

DUNN, O.J. (1964). Multiple comparison using rank sums. *Technometrics*, **6**, 241.

GRAHAM, S. (1964). Epidemiology of retinoids and cancer. *J. Natl Cancer Inst.*, **73**, 1423.

IP, C. (1982*a*). Vitamin E potentiates the prophylactic effect of selenium in chemically-induced mammary tumorigenesis. *Proc. Am. Assoc. Cancer Res.*, **23**, 70 (*abstract*).

IP, C. (1982*b*). Dietary vitamin E intake and mammary carcinogenesis in rats. *Carcinogenesis*, **3**, 1453.

KALBFLEISCH, J.D. & PRENTICE, R.L. (1979). *The statistical analysis of failure time data*. Wiley: New York.

KARK, J.D., SMITH, A.H., SWITZER, B.R. & HAMES, C.G. (1981). Serum vitamin A (retinol) and cancer incidence in Evans County, Georgia. *J. Natl Cancer Inst.*, **66**, 7.

KING, M.M. & OTTO, P. (1979). Null effect of BHA and -tocopherol on 7,12-dimethylbenz(a)anthracene-induced mammary tumours in rats fed different levels and types of dietary fat. *Proc. Am. Assoc. Cancer Res.*, **20**, 227 (*abstract*).

KOZIOL, A.J. & DONNA, A.M. (1981). A distribution free test for tumor growth curve analysis with application to an animal tumor immunotherapy experiment. *Biometrics*, **37**, 383.

LOTAN, R. (1980). Effects of vitamin A and its analogs (retinoids) on normal and neoplastic cells. *Biochim Biophys Acta*, **605**, 33.

McCAY, P.B., KING, M.M. & PITHA, J.V. (1981). Evidence that the effectiveness of antioxidants as inhibitors of 7,12-Dimethylbenz(a)anthracene-induced mammary tumours is a function of dietary fat composition. *Cancer Res.*, **41**, 3745.

McCORMICK, D.L., BURNS, F.J. & ALBERT, R.E. (1980). Inhibition of rat mammary carcinogenesis by short dietary exposure to retinyl acetate. *Cancer Res.*, **40**, 1140.

McCORMICK, D.L., BURNS, F.J. & ALBERT, R.E. (1981). Inhibition of Benzo(a)pyreneinduced mammary carcinogenesis by Retinyl Acetate. *J. Natl. Cancer Inst.*, **66**, 559.

McCORMICK, D.L. & MOON, R.C. (1982). Influence of delayed administration of retinyl acetate on mammary carcinogenesis. *Cancer Res.*, **42**, 2639.

MOON, R.C., GRUBBS, C.J., SPORN, M.B. & GOODMAN, D.G. (1977). Retinyl acetate inhibits mammary carcinogenesis induced by N-methyl-N-nitrosourea. *Nature*, **267**, 620.

MOON, R.C., MEHTA, R.G. & McCORMICK, D.L. (1983). Suppression of mammary cancer by retinoids. *Cancer: Etiology and Prevention*, 275.

NEWBERNE, P.M. & SUPHAKARN, V. (1983). Nutrition and cancer: A review, with emphasis on the role of vitamins C and E and Selenium. *Nutrition and cancer*, **5**, 107.

POLLIACK, A. & LEVIJ, I.S. (1969). The effect of topical vitamin A on papillomas and intraepithelial carcinomas induced in hamster cheek pouches with 9,10-Dimethyl-1.2-benzanthracene. *Cancer Res.*, **29**, 327.

QUANDER, R.V., LEARY, S.L., STRANDBERG, J.D., YARBOROUGH, B.A. & SQUIRE, R.A. (1985). Long-term effect of 2-Hydroxyethyl Retinamide on urinary bladder carcinogenesis and tumor transplantation in Fischer 344 rats. *Cancer Res.*, **45**, 5235.

ROMPPANEN, U., TUIMALA, R., PUNNONEN, R. & KOSKINEN, T. (1985). Serum vitamin A and E levels in patients with Lichen Sclerosus and carcinoma of the vulva – effect of oral etretinate treatment. *Ann. Chirurg. Gynaecol.*, **74**, 197, 27.

SCHMÄHL, D., KRÜGER, C. & PREISSLER, P. (1972). Versuche zur Krebsprophylaxe mit Vitamin A. *Arzneim. Forsch.*, **22**, 946.

SCHMÄHL, D. & HABS, M. (1978). Experiments on the influence of an aromatic retinoid on the chemical carcinogenesis in rats by Butyl-butanol-nitrosamine and 1,2-Dimethylhydrazine. *Drug Res.*, **28**, 1, 49.

SHAMBERGER, R.J. & RUDOLPH, G. (1966). Protection against cocarcinogenesis by antioxidants. *Experientia*, **22**, 116.

SHEKELLE, R.B., LIU, S., RAYNOR, W.J., JR & 6 others (1981). Dietary vitamin A and risk of cancer in the Western Electric study. *Lancet*, **ii**, 1185.

STONE, J.P., SHELLABARGER, C.J., & HOLTZMAN, S. (1984). The long-term inhibition of induced and spontaneous rat breast carcinogenesis by retinyl acetate: Interim results. *Proc. Am. Assoc. Cancer Res.*, **126**, 000 (*abstract*).

THOMPSON, H.J. & RONAN, A.M. (1983). Inhibition of 1-Methyl-1-Nitrosourea (MNU)-induced mammary tumorigenesis by Difluoromethylornithine (DFMO) and retinyl acetate (RA). *Proc. Am. Assoc. Cancer Res.*, 342 (*abstract*).

THOMPSON, H.J., HERBST, E.J. & MEEKER, L.D. (1986). Chemoprevention of mammary carcinogenesis: A comparative review of the efficacy of a polyamine antimetabolite, retinoids, and selenium. *J. Natl Cancer Inst.*, **77**, 595.

WATTENBERG, L.W. (1972). Inhibition of carcinogenic and toxic effects of polycyclic hydrocarbons by phenolic antioxidants and ethoxyamin. *J. Natl Cancer Inst.*, **48**, 1425.

WATTENBERG, L.W. (1978). Inhibition of chemical carcinogenesis. *J. Natl Cancer Inst.*, **60**, 11.

WELSCH, C.W., BROWN, C.K., GOODRICH-SMITH, M., CHIUSANO, J. & MOON, R.C. (1980). Synergistic effect of chronic Prolactin suppression and Retinoid treatment in the prophylaxis of N-Methyl-N-nitrosourea-induced mammary tumorigenesis in female Sprague–Dawley rats. *Cancer Res.*, **40**, 3095.

WELSCH, C.W. & DEHOOG, J.V. (1983). Retinoid feeding, hormone inhibition, and/or immune stimulation and the genesis of carcinogen-induced rat mammary carcinomas. *Cancer Res.*, **43**, 585.

ZILE, M.H., CULLUM, M.E., ROLTSCH, I.A., DEHOOG, J.V. & WELSCH, C.W. (1986). Effect of moderate vitamin A supplementation and lack of dietary vitamin A on the development of mammary tumours in female rats treated with low carcinogenic dose levels of 7,12-Dimethylbenz(a)anthracene. *Cancer Res.*, **46**, 3495.